# Rapid Glass Wool Enrichment of Glycopeptides for MALDI-MS Analysis of Immunoglobulin G Glycosylation in COVID-19 Samples

**DOI:** 10.1021/jasms.5c00205

**Published:** 2025-10-31

**Authors:** Yuye Zhou, Felicia Karlahag, Sophia Schedin Weiss, Sara Jamshidi, Lars Tjernberg, Åsa Emmer

**Affiliations:** † School of Engineering Sciences in Chemistry, Biotechnology and Health, Department of Chemistry, Division of Applied Physical Chemistry, Analytical Chemistry, 7655 KTH Royal Institute of Technology, 10044 Stockholm, Sweden; ‡ Division of Neurogeriatrics, Department of Neurobiology, Care Sciences and Society, Center for Alzheimer Research, Karolinska Institutet, 17164 Solna, Sweden; § Consultys SUISSE, Avenue de la gare 33, CH-1003 Lausanne, Switzerland

## Abstract

Glycosylation is the most common protein post-translational modification, affecting protein properties and functions. Abnormal variations in glycosylation are associated with diseases, e.g., coronavirus disease COVID-19. Matrix-assisted laser desorption/ionization mass spectrometry has been widely utilized for studying protein glycosylation, after proper purification of glycopeptides or glycans using hydrophilic interaction liquid chromatography (HILIC) or laboratory-synthesized hydrophilic materials. Here, glass wool tips were developed to enrich immunoglobulin G glycopeptides and applied in the analysis of COVID-19 patient samples as a proof-of-concept. A significant decrease in galactosylation was detected in the COVID-19 patient plasma sample compared to the reference sample. The tips developed in this work provided a cheap and simple enrichment alternative to commercial HILIC tips for studying protein glycosylation.

## Graphical abstract

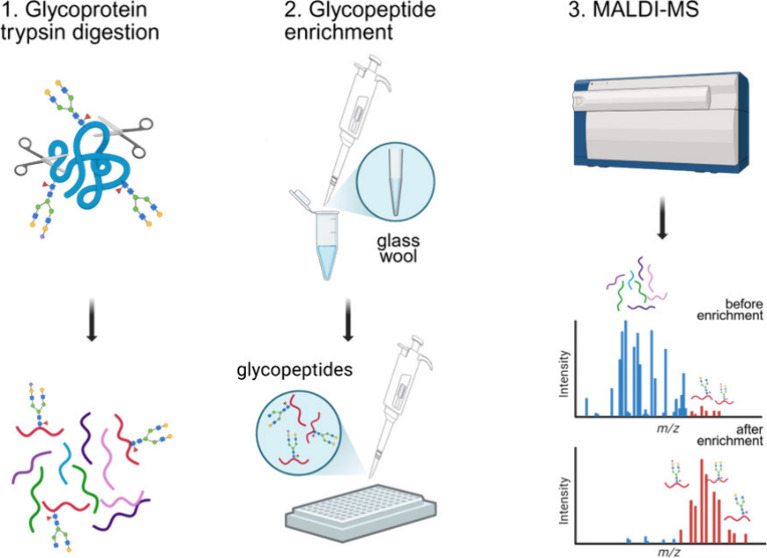

## Introduction

Protein glycosylation is one of the most important post-translational modifications (PTMs), conveying the physiological properties and biological activities of proteins, such as stability, structure, binding affinity, and immune defense.^1,2^ The study of glycosylation, in particular, N-linked glycosylation with carbohydrates attached to an asparagine residue, can lead to the discovery of biomarkers for diseases, such as cancers,^3,4^ inflammatory disease,^5,6^ Alzheimer’s disease,^7^ and coronavirus disease COVID-19.^8,9^ COVID-19, caused by severe acute respiratory syndrome coronavirus 2 (SARS-CoV-2), has killed about seven million people worldwide. Patients with severe COVID-19 have been characterized by increased inflammatory responses, such as increased levels of inflammatory cytokines, and alteration of immunoglobulin G glycosylation in the Fc domain.^9–11^ Decreases in galactosylation and sialylation have been reported related to patients with severe symptoms and play important roles in activating the lectin-initiated alternative complement pathway.^12,13^ Therefore, profiling the IgG N-glycome is crucial for studies aimed at understanding COVID-19 and other inflammatory diseases.

To study protein glycosylation, mass spectrometry techniques, including MALDI-TOF MS, are widely used.^14,15^ The analyses are often carried out on enzymatically digested glycoproteins to obtain information on both glycan structures and localization of glycosylation sites with the help of tandem MS. However, the presence of nonglycosylated peptides, salts, and impurities in the digest significantly suppresses the detection of glycopeptides due to the poor ionization efficiency of glycopeptides.^16^ Therefore, it is necessary to enrich and purify the glycopeptides from the digestion mixture and complicated sample matrices. For this purpose, many lab-synthesized materials based on different strategies including hydrophilic interaction liquid chromatography (HILIC),^17,18^ lectin chromatography,^19^ hydrazide chemistry,^20^ and boronic acid affinity have been described.^21^ However, most methods require a long preparation time and cautious control of the structure and functionalization of the materials. Therefore, the development of a fast, sensitive, and efficient method for the enrichment of glycopeptides based on easily obtained materials is highly demanded. Earlier, cellulose-based materials, including cotton wool and wood, have been suggested.^22,23^ Further, silica-based nanomaterials and modified silica gels have been used for glycopeptide enrichment.^24,25^ Cellulose-based materials are rich in hydroxyl groups (−OH) and highly hydrophilic interacting with glycopeptides, through hydrophilic interaction, partitioning, adsorption, and electrostatic interactions.^26^ For silica-based materials, silanol groups (≡SiOH) and siloxanes (≡Si—O—Si≡) on the surface offer the possibility to adsorb a wide range of analytes like proteins and peptides.^27,28^ Silanol groups can deprotonate at neutral pH, resulting in a negatively charged surface, and be positively charged at pH below 3,^29^ promoting hydrophilic interactions and electrostatic interactions with glycopeptides. Glass wool contains the same functional groups as silica gel and also provides a large surface area, bringing potential adsorption sites. Moreover, it is an inexpensive and easily available alternative. In this work, glass wool was introduced as a novel sorbent in micropipet tips for efficient and fast glycopeptide enrichment of tryptic digests of immunoglobulin G (IgG). Analysis of the COVID-19 patient samples was used as a proof-of-concept.

## Material and Methods

### Chemicals and Consumables

The human IgG standard samples were immunoglobulin G from human serum (56834–25MG), from Sigma-Aldrich (Stockholm, Sweden). Trypsin from the bovine pancreas (T1426), bovine serum albumin (BSA, A3059), acetonitrile (ACN), DL-dithiothreitol (D9779, DTT), iodoacetamide (I670–9, IAA), trifluoracetic acid (TFA), and ammonium bicarbonate (NH_4_HCO_3_) were also purchased from Sigma-Aldrich (Stockholm, Sweden). 2,5-Dihydroxybenzoic acid (DHB) was obtained from Bruker Daltonics (Bremen, Germany). The water used throughout the present work was purified with a Milli-Q Reference Water Purification System (Millipore SAS, Molsheim, France). Eppendorf Safe-Lock tubes (0.5 and 1.5 mL), Eppendorf Protein LoBind tubes (1.5 mL), and micropipette tips (Eppendorf, epT.I.P.S. standard/bulk 0.1–20 μL and 2–200 μL) used were all purchased from Sigma-Aldrich (Stockholm, Sweden). The glass wool used was PYREX Fiber Glass Roving (3950, Corning Glass Works, NY.).

### Trypsin Digestion of IgG Standard

An IgG standard solution was mixed with trypsin, NH_4_HCO_3_ buffer (pH = 8.0, final concentration 10 mM), and water in an Eppendorf lo-bind tube for digestion. IgG had a final concentration of 1 mg/mL in the solution, and the mixing ratio between IgG and trypsin was 30/1 (w/w). The mixture was incubated at 37 °C for 17 h in an Eppendorf ThermoMixer C (Hamburg, Germany) under vibration of 1000 rpm, and the digestion was then quenched at 75 °C for 5 min. Samples were stored at – 20 °C until enrichment and analysis. BSA was digested by the same method.

### Glycopeptide Enrichment Using Glass Wool Tips

A two mg portion of glass wool was weighed and transferred to a 200 μL micropipet tip and packed to a specified level with a blunt syringe needle (Figure S1 in Supporting Information). The outermost end of the micropipette tip (3 mm) was cut to remove the empty space. The tip was wetted with 100 μL of H_2_O five times and then preconditioned with 100 μL of loading solution five times. The sample solution was prepared by adding 5 μL of IgG digest (corresponding to 5 μg of IgG when using a concentration of 1 mg/mL) to different ratios of ACN, TFA, and H_2_O to get a total volume of 100 μL. To estimate the limit of detection, diluted IgG digest samples with concentrations of 0.025, 0.05, 0.1, and 0.5 mg/mL were also investigated. The sample was loaded into the tip by pipet aspiration 20 times. The tip was then washed with 100 μL of the washing solution. After this, the sample was eluted with 100 μL of elution solution by tip aspiration 5 times. The elution fraction was dried in an Eppendorf centrifugal vacuum concentrator 5301 (Hamburg, Germany) before it was reconstituted with 5 μL of 0.1% TFA in H_2_O before loading onto the MALDI plate. The solutions tested in the work for optimization are listed in Table S1. The conditions selected regarding selectivity, recovery, and for IgG from real human plasma analysis were the following: loading solution was 88% ACN/0.1% TFA in H_2_O, the sample solution was 88% ACN/0.01% TFA in H_2_O containing IgG digest, tip wash was carried out with 100 μL of 88% ACN/0.01% TFA (1 time) and 100 μL of 88% ACN/0.02% TFA (1 time), and finally the glycopeptides were eluted with 100 μL of H_2_O (Figure 1). To investigate the influence of the protein digest:glass wool ratio on the enrichment efficiency, the amount of glycopeptides loaded to the tip was varied. A volume of 5, 6, 7, and 8 μL of a digested IgG sample of 1 mg/mL was loaded to tips packed with 2 mg of glass wool. The amount of glycopeptides present in the nonbinding fraction was studied with MALDI-MS.

**Figure 1:**
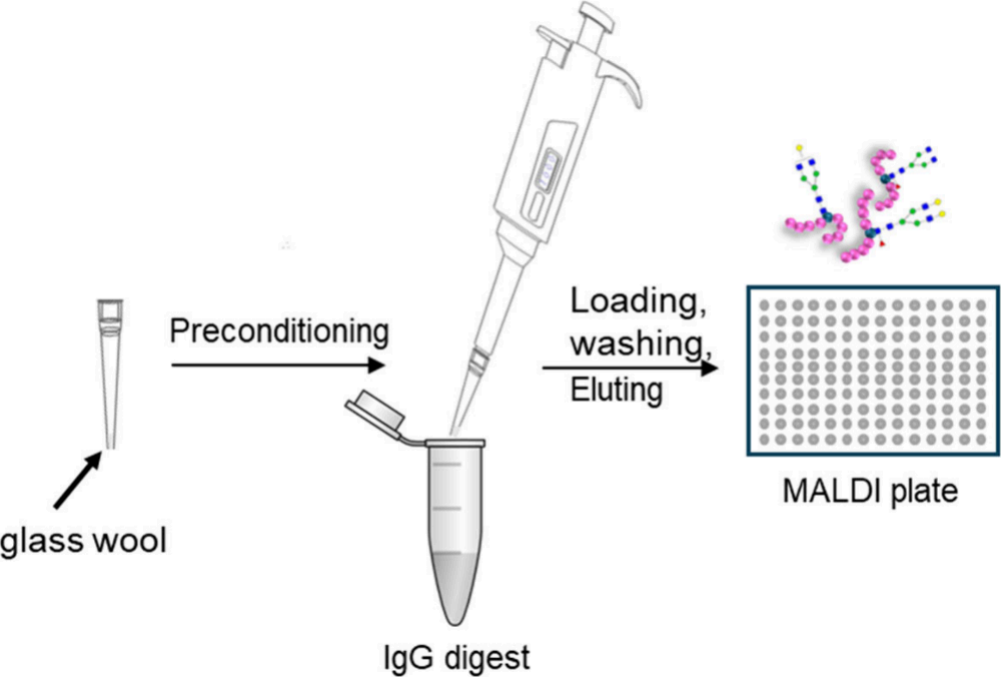
Schematic illustration of the glycopeptide enrichment process using a glass wool tip.

### Glycopeptide Enrichment Using HILIC Tips

For comparison, Hypersep HILIC tips (Thermo Fischer, 10–200 μL, Rockwood, TN, USA) were also used for glycopeptide enrichment. The enrichment conditions used here followed the instructions from the supplier and reference.^16^ Briefly, the tips were first wetted with 100 μL of H_2_O five times and then preconditioned with 100 μL of loading solution (86% ACN/1% TFA in H_2_O) 5 times. After this, the sample solution (5 μL of IgG digest in an ACN/TFA/H_2_O solution with the same ratio as the loading solution, total volume 100 μL) was loaded by pipet aspiration 20 times. The tip was washed with 100 μL of loading solution five times followed by elution with 100 μL of H_2_O.

### Biological Samples and Human Plasma IgG Preparation

The COVID-19 hospitalized patient plasma samples were obtained from the Karolinska Institute Biobank. The samples were stored at – 80 °C in the laboratory of Karolinska Institute having permission for the storage of samples of Biosafety Level-2 (BSL-2). The ethical permit (DNR 2020-05712) was approved by the Swedish Ethical Review Authority in Sweden for performing research on plasma samples derived from COVID-19 patients. In this work, four patient plasma samples were used. The commercial human plasma from pooled human blood (P9523-1 ML) was used as a reference sample (healthy plasma). Before sample preparation, all of the plasma samples were diluted 10 times with 20 mM Tris-HCl (pH 8.0) buffer and disinfected in 65 °C for 30 min. To isolate IgG from plasma, Protein A–Agarose (Protein A, P1406, Sigma-Aldrich, Sweden) was used. Ten mg of Protein A was swollen in 50 μL of binding buffer (20 mM Tris-HCl pH 8.0) for 30 min and then conditioned with 100 μL of binding buffer for 5 min in 1.5 mL LoBind tubes. The supernatant liquids were discarded after centrifugation using a Biofuge Pico microcentrifuge (Langenselbold, Germany). For all steps, centrifugation was carried out at 2400*g* for 3 min. Human plasma IgG preparation procedures refer to the supplier and a previously reported method.^30^ Briefly, 50 μL of diluted and disinfected plasma was loaded onto the preconditioned Protein A and mixed for 30 min in a ThermoMixer at 1000 rpm. The supernatant was discarded and IgG-bound Protein A was washed two times with 500 μL of 20 mM Tris-HCl + 0.5 M NaCl, pH 8.0 for 30 min under vibration of 1000 rpm. IgG was eluted with 200 μL of 0.1 M Glycine-HCl, pH 2.5. Collected IgG was neutralized with 1 M Tris-HCl, pH 8.5.

### Trypsin Digestion of IgG Obtained from COVID-19 Patients and Reference Human Plasma Samples

Before trypsin digestion, IgG obtained from 5 μL of plasma was reduced with DTT (final concentration: 10 mM) at 60 °C for 30 min and alkylated with IAA (final concentration: 25 mM) in the dark at 37 °C for 30 min in a ThermoMixer. After reduction and alkylation, the sample solution was desalted using Pierce C18 Tips. The desalted IgG sample was dried and reconstituted in 10 μL of H_2_O for trypsin digestion. After that, 2 μg of trypsin was added to the sample for digestion. The buffer used here for digestion was also 10 mM NH_4_HCO_3_ (pH 8.0).

### Glycopeptide Enrichment of Glycopeptides from COVID-19 Patients and Reference Human Plasma IgG Using Glass Wool Tips

A ten μL portion of IgG digest was mixed with ACN, TFA, and H_2_O to get a total volume of 100 μL (88% ACN/0.01% TFA). The method used was almost the same as the optimized method for glycopeptide enrichment from the IgG standard sample, except for using 30 times pipet aspiration for sample loading.

### MALDI-TOF–MS Analysis of IgG Glycopeptides

A 0.5 μL sample was applied on a MALDI ground steel plate. 0.5 μL of matrix was applied on the dried sample droplets. For each sample, several spots were prepared. After crystallization, sample spots were analyzed on an UltrafleXtreme MALDI TOF/TOF mass spectrometer (Bruker Daltonics, Bremen, Germany), which was equipped with a Smartbeam-II laser (355 nm, UV). Twenty mg/mL DHB in 30 ACN/70 0.1% TFA v/v (TA 30) was used as matrix, and samples were analyzed in positive reflectron MS mode, with laser intensity set to 80% and 5000 shots summed. Random walk (partial sample) with 50 shots at raster spot and a diameter limit of 800 μm was used to get homogeneous sampling. The masses of obtained IgG glycopeptides were compared with masses presented in the literature.^16,23^ For more accurate glycopeptide identification, MSMS could also be performed to gain more detailed information and decreased risk of erroneous interpretations. Even so, due to the simple sample system of purified IgG and a single glycosylation site in each IgG heavy chain, the determination of glycoforms can be achieved using MS lists and previously reported glycosylation patterns.^31^

## Results and Discussion

### Optimization of Loading, Washing, and Elution Solutions

The optimization of glycopeptide enrichment using glass wool was carried out on tryptic digests of commercial human serum IgG (IgG standard). The loading, washing, and elution solutions, respectively, were chosen to minimize the occurrence of glycopeptides in nonbinding fractions and nonglycopeptides in elution fractions and to improve the detection of glycopeptides in the elution fractions. The hydrophilic interaction and electrostatic interaction between the glass wool and glycopeptides can be affected by adjusting the proportion of organic solvent and acid in the solutions. The optimization aims to maximize the yield of enriched glycopeptides while minimizing the extraction of potentially interfering nonglycopeptides.

The proportion of ACN in the loading solution could affect the partitioning of both glycopeptides and nonglycopeptides between the sample solution and the adsorption materials.^16^ TFA in the loading solution acts as an ion-pairing agent, protonating glycopeptides and neutralizing the highly charged nonglycopeptides. Loading solutions containing 85% ACN/0.1% TFA, 88% ACN/0.1% TFA, and 90% ACN/0.1% TFA were applied, and the samples were prepared to contain the same proportion of ACN as the loading solutions. These loading solutions, as the assessed washing and elution solutions, were chosen based on earlier experience.^16^ Only minor differences could be seen upon comparison of the results. Further, there were only low amounts of glycopeptides present in the nonbinding fractions in all three cases (Figure S2), indicating the successful binding of most of the IgG glycopeptides to the glass wool. The glycopeptides bound to the glass wool using the three different loading solutions were eluted with H_2_O (Figure S3). Some differences could be observed in the results with 90% ACN showing fewer peaks and lower intensities. Due to the earlier mentioned reports on the galactosylation effect of COVID-19 severity, the S/N values for the six main glycopeptides with differences in galactosylation (*m*/*z* 2602, 2634, 2764, 2796, 2926, and 2958) were compared (Table S2). The spectra obtained using a loading solution containing 88% ACN resulted in a slightly decreased detection of nonglycopeptides compared to that of 85% ACN. Thus, the ACN proportion of 88% in the loading solution, showing slightly higher selectivity for glycopeptides, was selected for further optimization. Considering TFA, loading solutions containing 0.1%, 1%, and 2% TFA were compared. Higher amounts of glycopeptides were present in the nonbinding fractions when 1% TFA or 2% TFA was added to the loading solution (Figure S4). Therefore, a loading solution with 88% ACN/0.1% TFA was used for further studies to avoid nonglycopeptides in the final samples.

Different washing and elution solutions were also investigated. Two elution solutions were compared, H_2_O and 0.1% TFA. The spectra obtained when eluting with H_2_O contained fewer nonglycopeptides compared to that of 0.1% TFA. Furthermore, the S/N values for the glycopeptides were higher for H_2_O eluted samples than that of 0.1% TFA eluted samples (Figure S5, Table S3). Regarding the washing solution, the effect of the proportion of TFA (88% ACN, 88% ACN/0.01% TFA, 88% ACN/0.02% TFA, and 88% ACN/0.03% TFA) was investigated. We observed that washing solutions containing higher proportions of TFA (0.02% or 0.03% TFA) could improve the separation of nonglycopeptides from glycopeptides (Figure S6 and Figure S7). Nevertheless, the S/N values of the main glycopeptides decreased when a higher proportion of TFA was applied to the washing solution (Table S4). Therefore, it is recommended to wash the tips with 100 μL of 88% ACN/0.01% TFA followed by 100 μL of 88% ACN/0.02% TFA before elution to remove contaminating substances. In summary, the recommended solutions to be used in the different steps for glycopeptide enrichment using glass wool tips are as follows: (1) Preconditioning: H_2_O and loading solution (88% ACN/0.1% TFA in H_2_O). (2) Sample solution: IgG digested in 88% ACN and 0.01% TFA in H_2_O. (3) Washing solution: 88% ACN/0.01% TFA in H_2_O and 88% ACN/0.02% TFA in H_2_O. (4) Elution: H_2_O.

To optimize the ratio between the weight of glass wool and the loading amount of IgG digest, the effect of the protein digest:glass wool ratio on the presence of glycopeptides in the nonbinding fractions is illustrated in Figure S8. It can be observed that almost no glycopeptides could be detected in the nonbinding fraction when 5 μg of IgG digest was loaded onto 2 mg of glass wool for glycopeptide enrichment. Increased intensity of glycopeptides can be observed in the nonbinding fraction with increased loading amount of IgG digest. Thus, we recommend a ratio of 5 μg of protein digest to 2 mg of glass wool for glycopeptide enrichment.

### Comparison of Glass Wool Tips and Commercial HILIC Tips for Glycopeptide Enrichment

After optimization of the procedure, glass wool tips were compared to commercial HILIC tips for IgG glycopeptide enrichment from IgG standard samples. There were only a few nonglycopeptides detected (*m*/*z* less than 2200) in the elution fractions after glycopeptide enrichment using either material. Results for enriched glycopeptides using the two tips are shown in Figure 2 and listed in Table S5 with structure information for the glycans. There are 18 and 19 glycopeptides enriched from IgG digest using a glass wool tip and HILIC tip, respectively. Eleven glycopeptides from IgG1 and seven glycopeptides from IgG2 were enriched by glass wool tips, similar to the numbers enriched by HILIC tips, which were 12 for IgG1 and seven for IgG2. The higher numbers and also higher intensities of glycopeptides observed from IgG1, compared to IgG2, are probably due to a higher proportion of IgG1 than IgG2 in human serum. The S/N values of the six main glycopeptides with differences in galactosylation are shown in Table S6. Glycopeptides enriched by glass wool tips generally showed slightly lower S/N values compared to those from HILIC tips, probably due to the lower hydrophilicity of glass wool. Nevertheless, glass wool tips provide a much cheaper alternative to commercial HILIC tips for IgG glycopeptide enrichment and galactosylation analysis.

**Figure 2:**
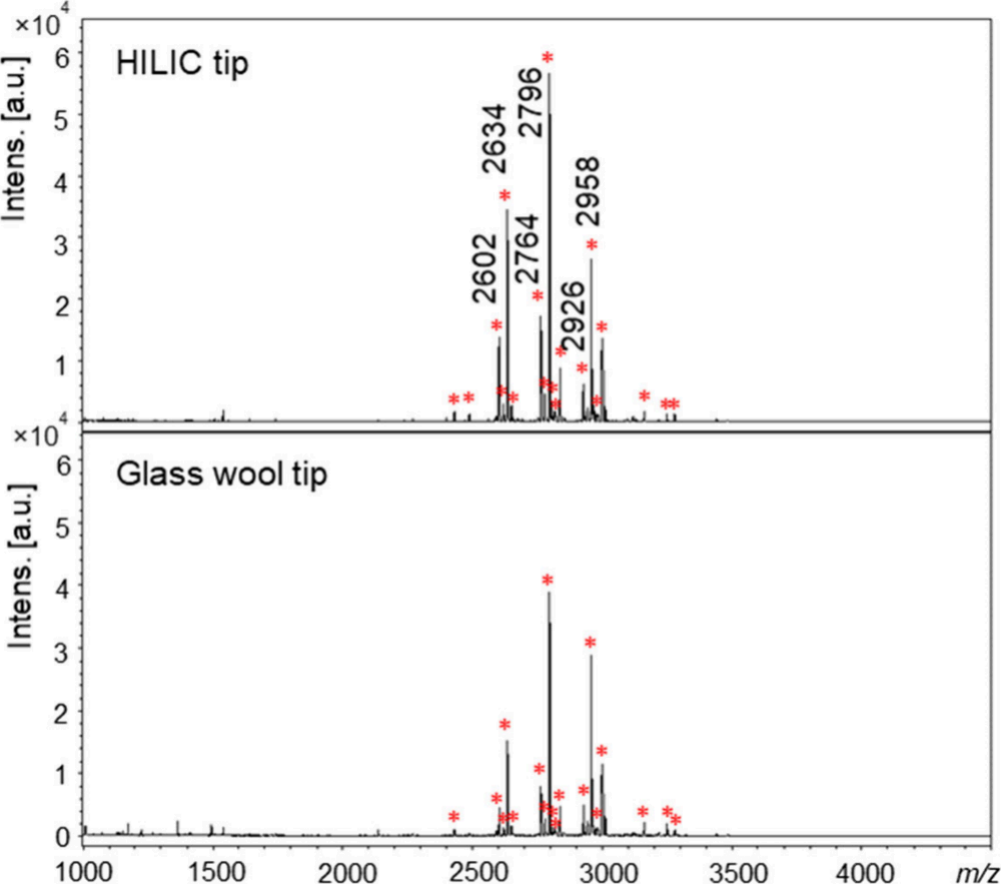
MALDI-TOF mass spectra of enriched glycopeptides using HILIC or a glass wool tip. Enriched glycopeptides are marked with red stars.

It could be observed from Figure 2 and Table S5 that only one glycopeptide with sialic acid (*m*/*z* 3249) was detected in both samples, which is due to the low ionization efficiency and instability of sialylated glycopeptides in an acidic environment using the positive mode. To assess if glass wool could be used to enrich sialylated glycopeptides, the enriched sample was also analyzed in negative mode. Three sialylated glycopeptides could be enriched using glass wool (Figure 3). Besides the low level of sialylation, the human IgG standard sample also showed low abundance of afucosylation (e.g., glycopeptides with *m*/*z* 2618, 2650, 2780, 2812) and presence of bisecting N-acetylglucosamine (e.g., glycopeptides with *m*/*z* 2805, 2837, 2967, 2999). The main glycopeptides detected from human IgG were the six fucosylated glycopeptides with a variation in galactosylation: *m*/*z* 2602 (from IgG2) and 2634 (from IgG1) without galactose (G0F), *m*/*z* 2764 (from IgG2) and 2796 (from IgG1) with one galactose (G1F), and *m*/*z* 2926 (from IgG2) and 2958 (from IgG1) with two galactoses (G2F). Therefore, for the following analysis, these six glycopeptides were studied.

**Figure 3:**
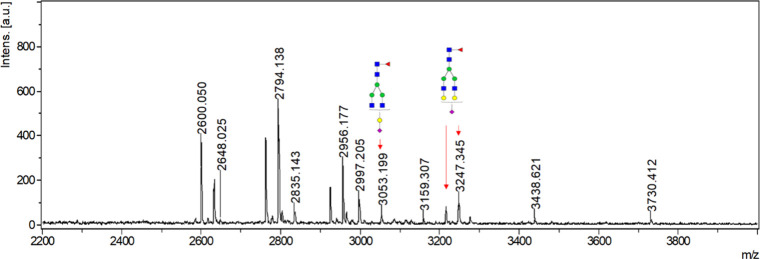
MALDI-TOF mass spectrum of glass wool enriched glycopeptides detected under negative mode. Sialylated glycopeptides are marked with red arrows.

### Reproducibility, Limit of Detection, Selectivity, and Recovery Using Glass Wool Tips

To assess the reproducibility of glass wool, enrichment experiments on IgG digests were carried out on different days. The data are shown in Table S6. Due to the intrinsic low capability in quantification of MALDI-MS, intensities and S/N usually vary from spot to spot. Variability can be observed between MALDI sample spots and replicates (by standard deviation) and days in absolute S/N values. This variation can also be observed in the HILIC-enriched replicates. On the other hand, the relative abundances of each glycoform remained very similar between days, suggesting a good reproducibility for relative quantification of glycosylation using the proposed method.

To investigate the limit of detection using glass wool for IgG glycopeptide enrichment, we loaded IgG digests with various concentrations. As shown in Table S7, the six targeted glycopeptides could be detected in enriched samples down to a concentration of 0.05 mg/mL (330 fmol), but with a concentration of 0.025 mg/mL, the detectability was worse, especially for the glycopeptide with *m*/*z* 2926 (Figure S9). Therefore, we recommend a sample concentration of higher than 0.05 mg/mL for this workflow.

To further investigate the glycopeptide enrichment performance of glass wool tips, we assessed the selectivity and recovery. To investigate the selectivity, sample mixtures of 5 μL of IgG digest and 5 μL of BSA digest (1/1, 1/3, 1/5, and 1/10 w/w) were loaded onto the tips. It could be observed from the spectra using 1/1 w/w ratio (Figure S10) that most nonglycopeptides could be separated from the glycopeptides. However, the proportion of BSA influenced the detectability of the glycopeptides, and at higher amounts of albumin present in the sample mixture, the detection of IgG glycopeptides was not as good as that in the pure IgG sample (Figure S10 and Table S8). Therefore, it is recommended to deplete the albumin before analyzing patient samples. In typical IgG glycosylation analysis from human blood plasma, the steps involve IgG purification, enzymatic digestion or release of N-glycans, and glycopeptide enrichment/glycan derivatization.^32^ An IgG purification step is usually carried out with protein A or protein G, to isolate IgG from other plasma proteins including albumin and other glycoproteins. In this case, after glycopeptide enrichment, it is assured that glycopeptides originate from IgG rather than other glycoproteins.

To study the recovery of IgG glycopeptides from the glass wool after enrichment, enriched IgG glycopeptides were collected and reenriched using glass wool tips. Glycopeptides after a single enrichment cycle and after two enrichment cycles were collected, and the same amounts of internal standard (IS) were added to each sample (Figure S11). The IS used was a synthesized IgG1 glycopeptide (EEQYN­(GlcNAc)­STYR, *m*/*z* 1392). The area ratios between the main IgG1 glycopeptides (*m*/*z* 2634, 2796, and 2958) and the IgG1 IS were calculated for one and two enrichment cycles. The recovery for each glycopeptide was calculated using Equation S1. Obtained recoveries after two cycles were 59%, 81%, and 100% for IgG1 glycopeptides with *m*/*z* of 2634, 2796, and 2958, respectively (Table S9). The recoveries increased with the size of the glycans, probably due to the increased interaction between the glass wool and the glycopeptides with larger glycans.

### Analysis of COVID-19 Patient Plasma Samples

After the methods for human IgG standard samples were evaluated, glass wool tips were applied to enrich glycopeptides from COVID-19 patient plasma samples and compared with reference plasma samples. A mixture of plasma from four hospitalized patients with COVID-19 (samples obtained from Karolinska Institute Biobank) was used for IgG isolation using protein A agarose, trypsin digestion, and glycopeptide enrichment using glass wool. Human plasma purchased from Sigma-Aldrich was used as reference plasma. Both plasma samples were treated in the same way. Enriched glycopeptides from patient and reference plasma samples are shown in Figure 4.

**Figure 4:**
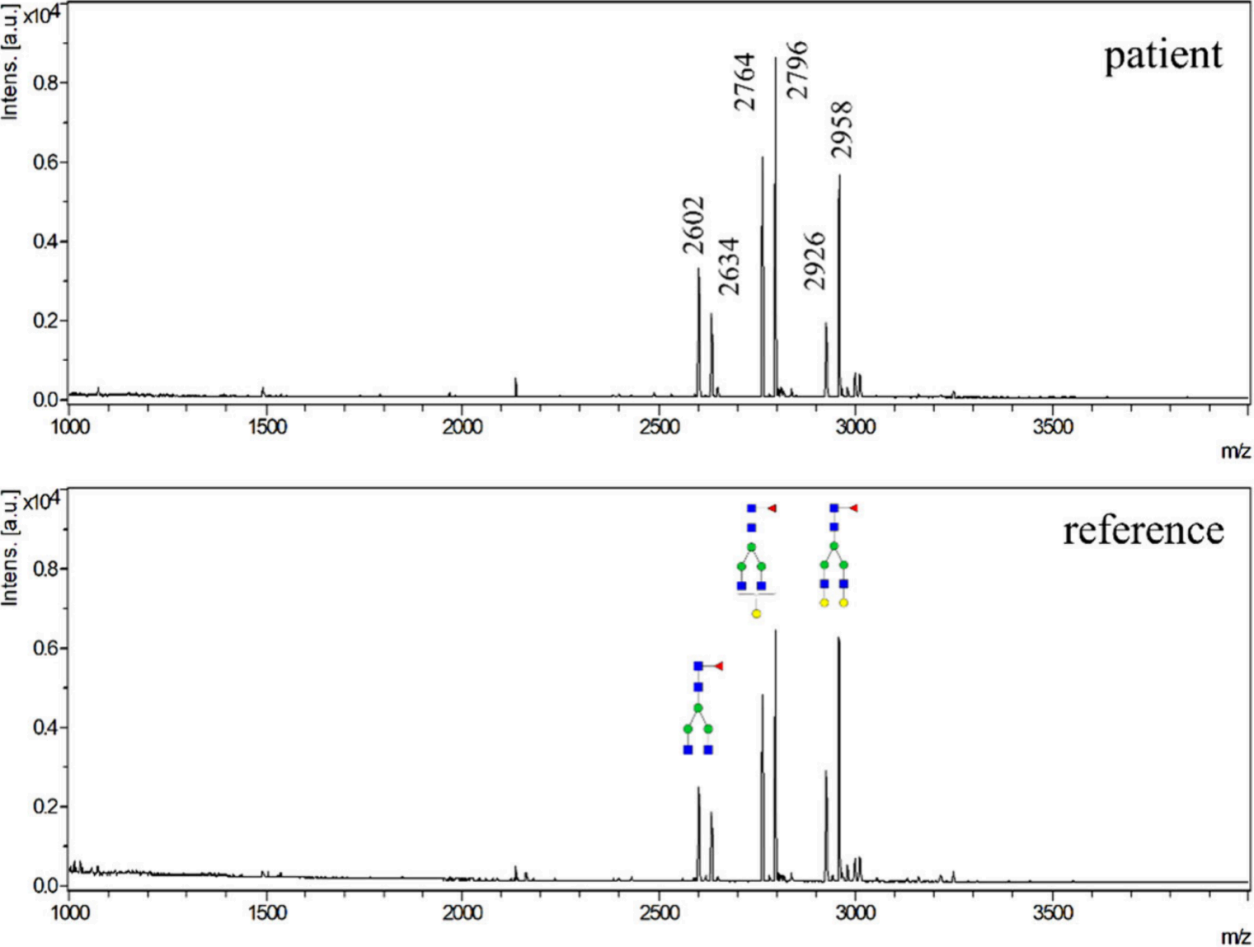
MALDI-TOF mass spectra of IgG glycopeptides enriched from a sample from the respiratory system of COVID-19 hospitalized patients and a reference plasma sample.

The main peaks were G0F, G1F, and G2F; the abundance of afucosylated and sialylated glycopeptides was low for both samples. Instead, the change in galactosylation in the sample from hospitalized patients compared to the reference sample was investigated. To assess the differences in IgG galactosylation between the COVID-19 patient sample and the reference sample, the relative proportions of the three glycoforms were compared based on the linear correlation between IgG glycoform abundance and MALDI-TOF MS response.^33^ The proportion of a certain glycoform was calculated by the area ratio between this glycoform and the sum of the three studied glycoforms. For example, the proportion of G0F was calculated by the following equation: P_G0F_ = Area_G0F_/(Area_G0F_ + Area_G1F_ + Area_G2F_) × 100%. Calculated proportions are shown in Figure 5 and Table S10. A decrease in G2F and an increase in G0F and G1F, could be observed for both IgG1 and IgG2 in the patient sample compared to the reference sample. The results agree with results presented in two recent publications.^12,13^ Even though the study is limited, the results obtained illustrate the potential of the use of glass wool tips in glycosylation studies. In the future use of this enrichment method, the use of internal standards could be considered. This may aid in quantification and increase the reliability of the results. However, internal standards for glycopeptides could be difficult and expensive to acquire.

**Figure 5:**
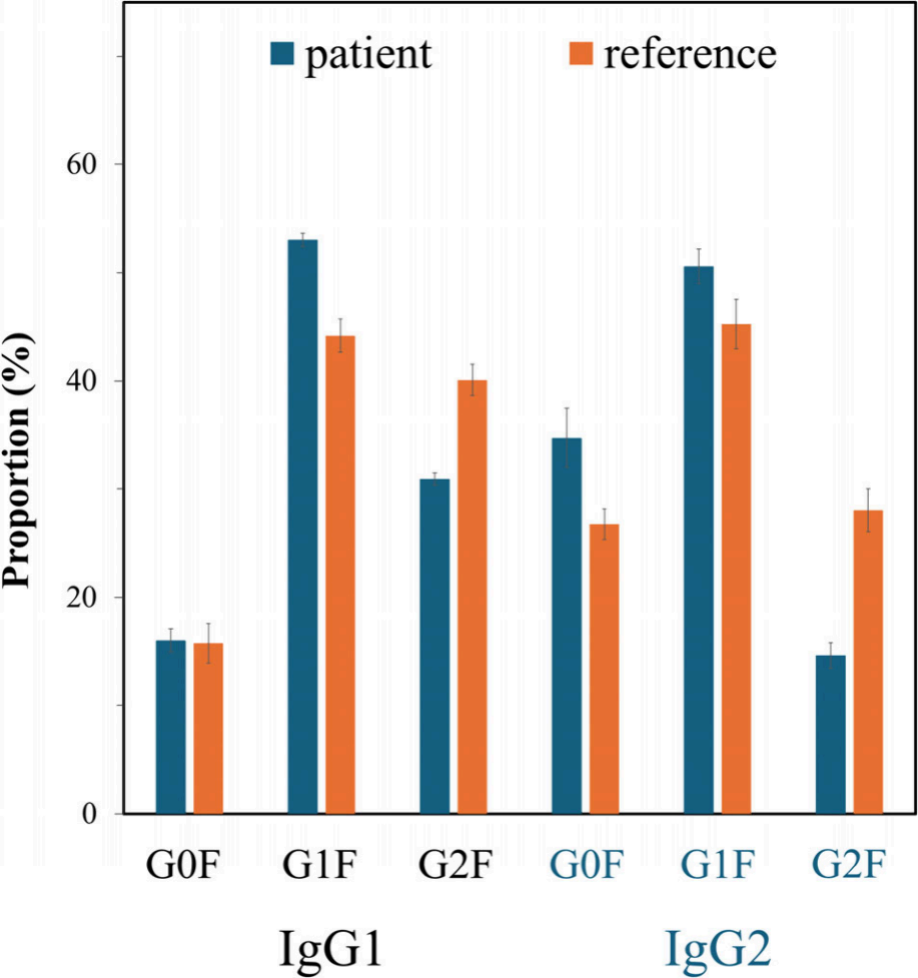
Proportions of G0F (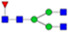), G1F (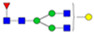), and G2F (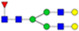) in IgG1 and IgG2 glycopeptides enriched from the COVID-19 patient sample and reference plasma sample. Blue square: N-Acetylglucosamine, red triangle: fucose, green circle: mannose, yellow circle: galactose. Data is represented as mean ± SD, *n* = 12, ns: no significance, ****p < 0.0001.

## Conclusions

The use of glass wool as an inexpensive and easily accessible affinity material for glycopeptide enrichment was developed and optimized. The glass wool tips showed performance comparable to that of commercially available HILIC tips, especially for less hydrophilic glycopeptides. Using negative mode mass spectrometry after enrichment with glass wool, we improved the detection of sialylated glycopeptides. The glass wool tips were applied to study the change in galactosylation in the COVID-19 samples as a proof-of-concept. A decrease in the more galactosylated G2F and an increase in G0F and G1F were observed in the patient sample compared to the reference sample. Though, it should be pointed out that the sample number was limited, and the results were not statistically verified. In summary, the glass wool tips provided a simple and fast alternative to commercial HILIC tips for the enrichment of glycopeptides from patient samples at a much lower cost.

## Supplementary Material



## Notes

Additional experimental results including image of packed tips, MALDI-MS spectra and tables of results from optimization experiments, and the equation for recovery calculations (PDF)
