# Supplementary material for: Rapid Glass Wool Enrichment of Glycopeptides for MALDI-MS Analysis of Immunoglobulin G Glycosylation in COVID-19 Samples
Source: J Am Soc Mass Spectrom. 2025 Oct 31;36(12):2615–22. doi: 10.1021/jasms.5c00205 (PMC12679630; doi:10.1021/jasms.5c00205)
Supplement: Supplementary file 1 [file js5c00205_si_001.pdf]

Supporting information

Rapid glass wool enrichment of glycopeptides for MALDI-MS analysis of Immunoglobulin G glycosylation in COVID-19 samples

Yuye Zhou,[1, ‡] Felicia Karlahag,[1, ‡] Sophia Schedin Weiss,[2] Sara Jamshidi,[1, 3] Lars Tjernberg,[2]

*Åsa Emmer [1,] **

1) School of Engineering Sciences in Chemistry, Biotechnology and Health, Department of Chemistry, Division of Applied Physical Chemistry, Analytical Chemistry, KTH Royal Institute of Technology, 10044 Stockholm, Sweden

2) Division of Neurogeriatrics, Department of Neurobiology, Care Sciences and Society, Center for Alzheimer Research, Karolinska Institutet, 17164 Solna, Sweden

3) Consultys SUISSE, Avenue de la gare 33, CH-1003 Lausanne, Switzerland

Corresponding Author

* Åsa Emmer, School of Engineering Sciences in Chemistry, Biotechnology and Health, Department of Chemistry, Division of Applied Physical Chemistry, Analytical Chemistry, KTH Royal Institute of Technology, 10044 Stockholm, Sweden. https://orcid.org/0000-0002-3444-9987, e-mail: aae@kth.se.

Author Contributions

‡These authors contributed equally.

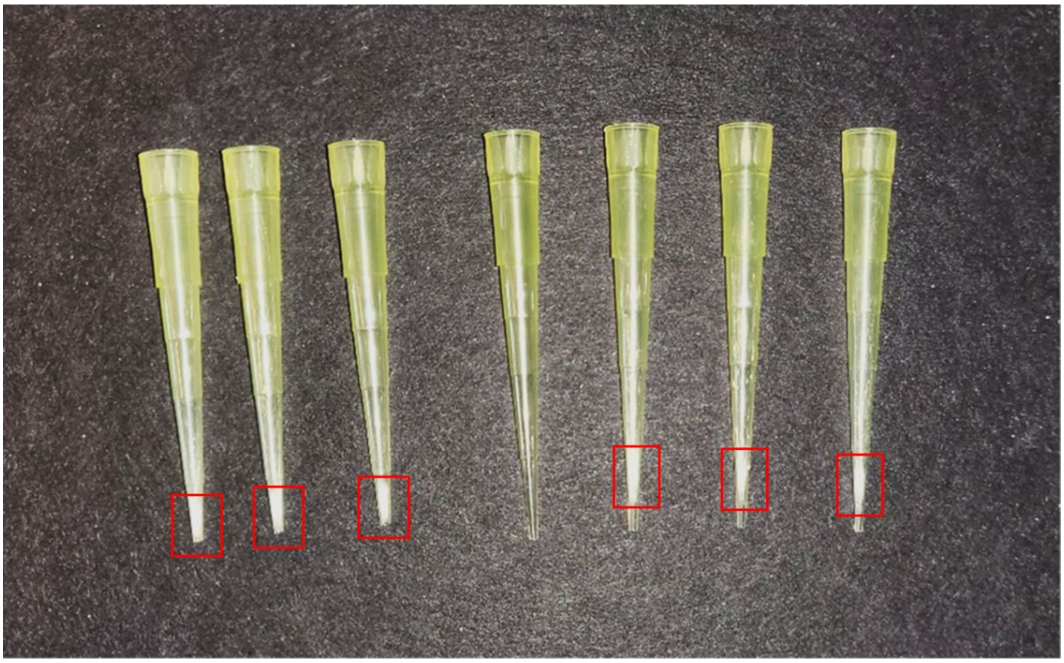

Figure S1. Glass wool tips, 200 µL micropipette tips packed with 2 mg glass wool. Glass wool is highlighted with red boxes. Left: three packed and cut tips, middle: non-packed tip, right: three packed but not cut tips.

Table S1. Loading solutions, washing solutions, and elution solutions tested.

	**Loading solution**	**Sample solution**	**Washing solution**	**Elution solution**
Loading solution optimization (ACN proportion)	85% ACN/0.1% TFA	5 µg IgG digest in 85% ACN	85% ACN (8 times)	H_2O
	88% ACN/0.1% TFA	5 µg IgG digest in 88% ACN	88% ACN (8 times)	
	90% ACN/0.1% TFA	5 µg IgG digest in 90% ACN	90% ACN (8 times)	
Loading solution optimization (TFA proportion)	88% ACN/0.1% TFA	5 µg IgG digest in 88% ACN	88% ACN (5 times)	H_2O
	88% ACN/1% TFA			
	88% ACN/2% TFA			
Elution solution optimization	88% ACN/0.1% TFA	5 µg IgG digest in 88% ACN	88% ACN (8 times)	H_2O
				0.1% TFA
Washing solution optimization	88% ACN/0.1% TFA	5 µg IgG digest in 88% ACN/0.01% TFA	88%ACN (3 times)	H_2O
			88%ACN/0.01%TFA (3 times)	
			88%ACN/0.02%TFA (3 times)	
			88%ACN/0.03%TFA (3 times)	
			88%ACN/0.02%TFA (1 time)	
			88%ACN/0.03%TFA (1 time)	
			88%ACN/0.01%TFA (1 time) and 88%ACN/0.02%TFA	

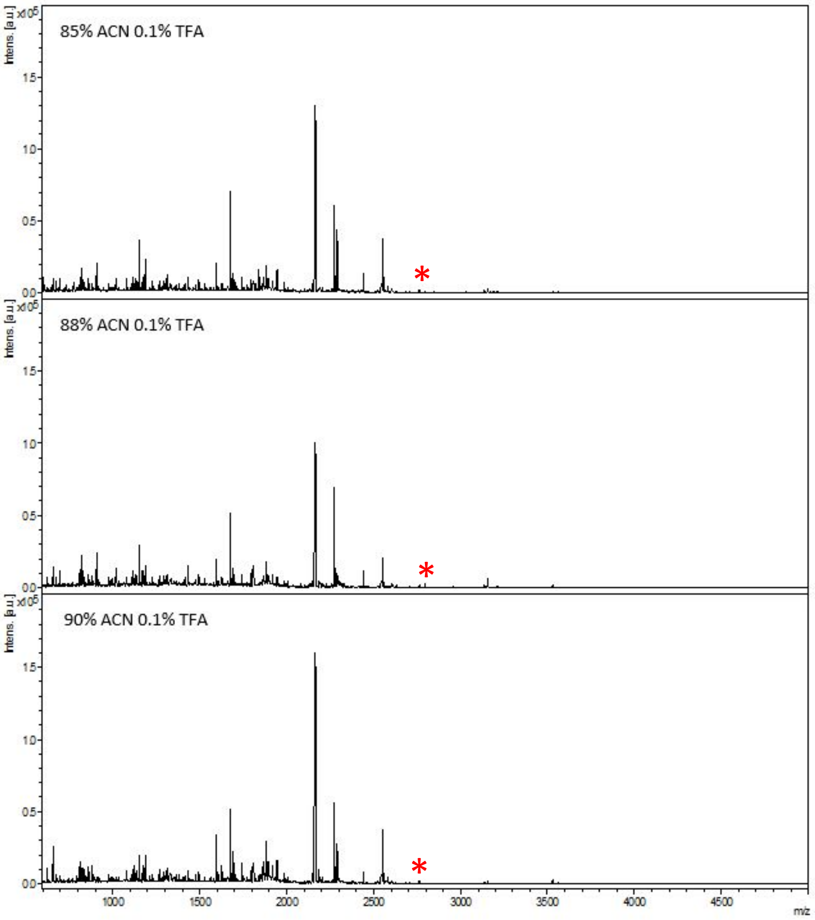

Figure S2. MALDI-TOF mass spectra of non-binding fractions from the enrichment procedure using loading solutions with different proportions of ACN, and 0.1% TFA in H_2O. Glycopeptides are marked with red stars. Almost no glycopeptides were present in the non-binding fractions.

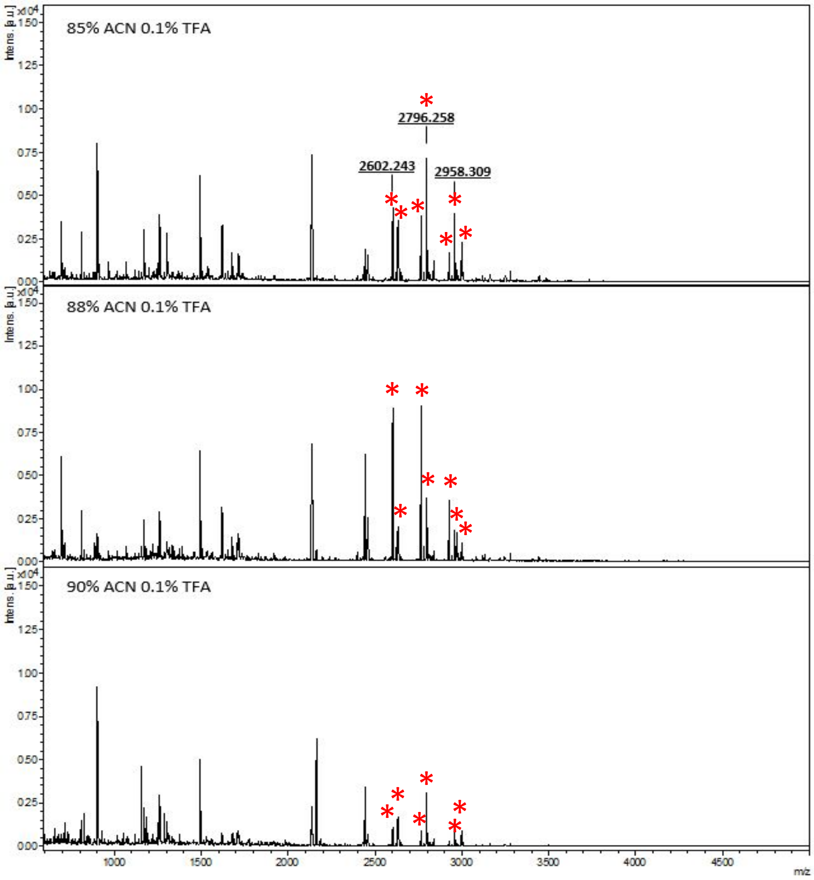

Figure S3. MALDI-TOF mass spectra of elution fractions from the enrichment procedure using loading solutions with different proportions of ACN, and 0.1% TFA in H_2O. Glycopeptides are marked with red stars.

Table S2. Average S/N values of the main glycopeptides detected in the elution fractions using three different ACN proportions. n = 5, from two replicates. Data are presented as "mean ± standard deviation". The structural difference between glycopeptides with m/z 2602/2634, 2764/2796, and 2926/2958 is that two phenylalanine entities have been exchanged for tyrosine.

m/z	Glycan	85% ACN	88% ACN	90% ACN
**2602**	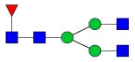	57.20 ± 11.69	69.60 ± 40.09	48.40 ± 22.19
**2634**		47.80 ± 15.21	39.40 ± 11.65	30.60 ± 6.43
**2764**	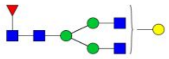	74.60 ± 22.92	79.80 ± 45.09	45.20 ± 22.61
**2796**		104.20 ± 33.73	77.00± 22.66	52.80 ± 12.32
**2926**	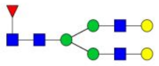	36.00 ± 10.22	35.40 ± 19.31	18.60 ± 9.15
**2958**		59.60 ± 18.81	43.40 ± 15.13	27.60 ± 6.80

■ N-acetylglucosamine (GlcNAc) ● Mannose ○ Galactose ◄ Fucose

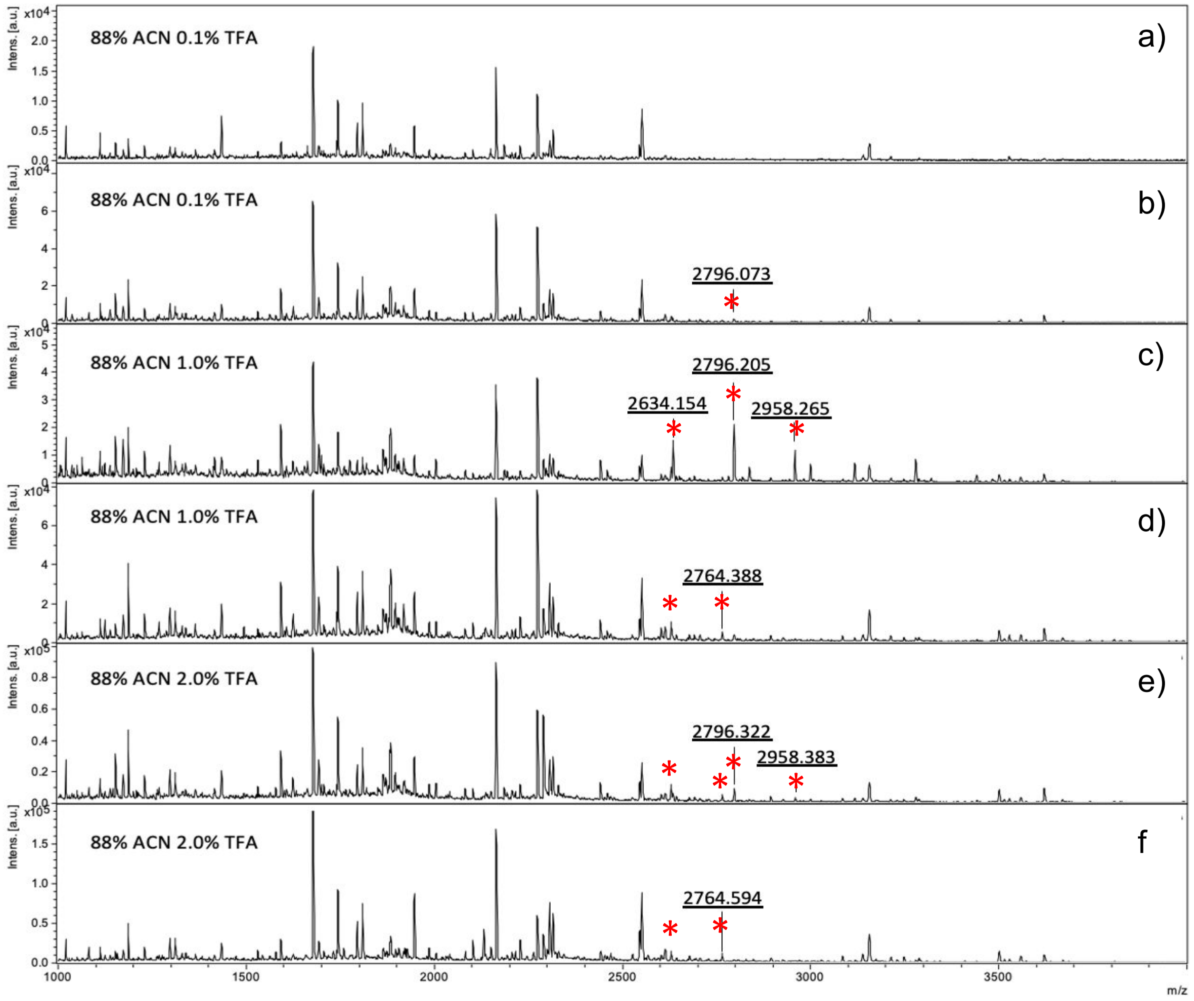

Figure S4. MALDI-TOF mass spectra of non-binding fractions from the enrichment procedure using loading solution with a) and b) 88% ACN 0.1% TFA, c) and d) 88% ACN and 1.0% TFA, e) and f) 88% ACN and 2.0% TFA. Glycopeptides are marked with red stars.

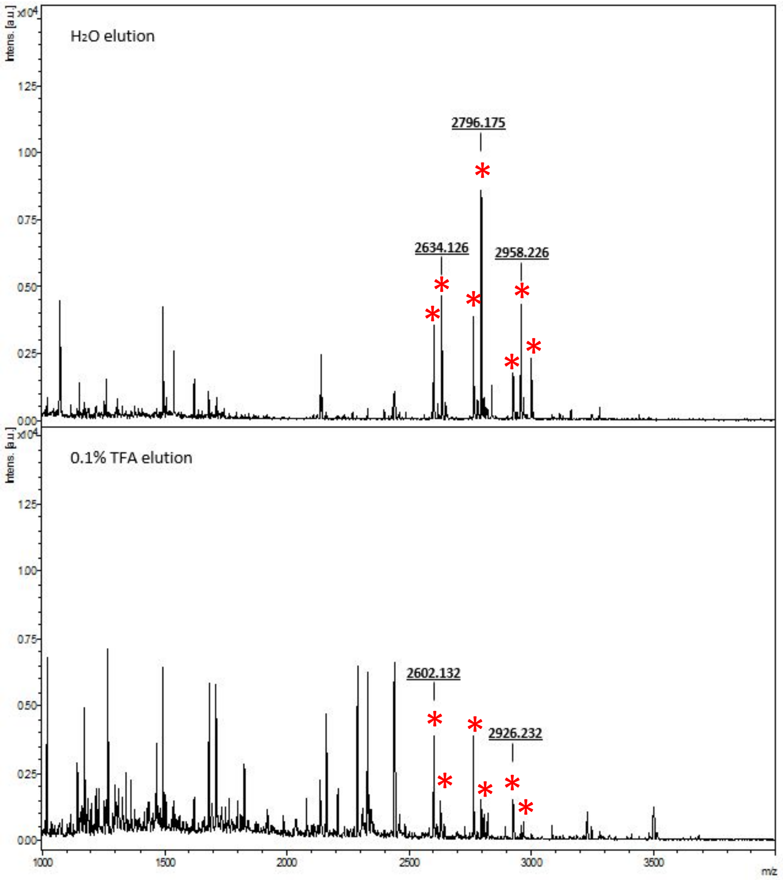

Figure S5. MALDI-TOF mass spectra of elution fractions using H_2O (top) and 0.1% TFA (bottom) as elution solution. Loading solution: 88% ACN/0.1% TFA. Glycopeptides are marked with red stars.

Table S3. Average S/N values of the six main glycopeptides detected in the elution fractions using different elution solutions. n = 5, from two replicates. Data are presented as "mean ± standard deviation".

m/z	H_2O elution	0.1% TFA elution
**2602**	70.40 ± 39.46	47.40 ± 26.18
**2634**	43.60 ± 14.10	21.00 ± 13.51
**2764**	83.20 ± 42.27	53.00 ± 31.91
**2796**	86.80 ± 27.36	35.00 ± 33.87
**2926**	37.20 ± 17.88	22.80 ± 15.01
**2958**	48.80 ± 16.57	22.60 ± 15.42

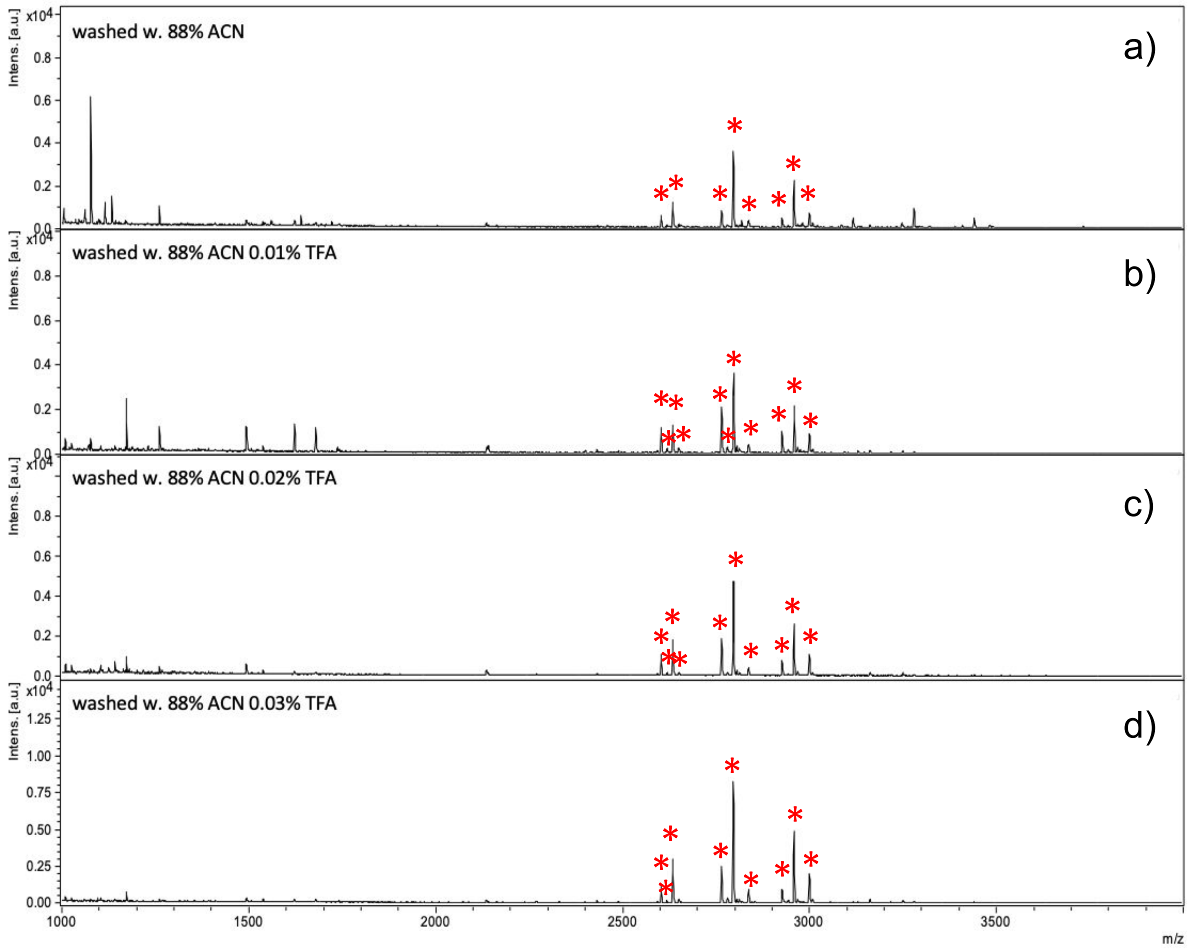

Figure S6. MALDI-TOF mass spectra of elution fractions with different proportions of TFA in the washing solution: a) 88% ACN, b) 88% ACN/0.01% TFA, c) 88% ACN/0.02% TFA, and d) 88% ACN/0.03% TFA. Each sample was washed three times with 100 µl washing solution. Glycopeptides are marked with red stars.

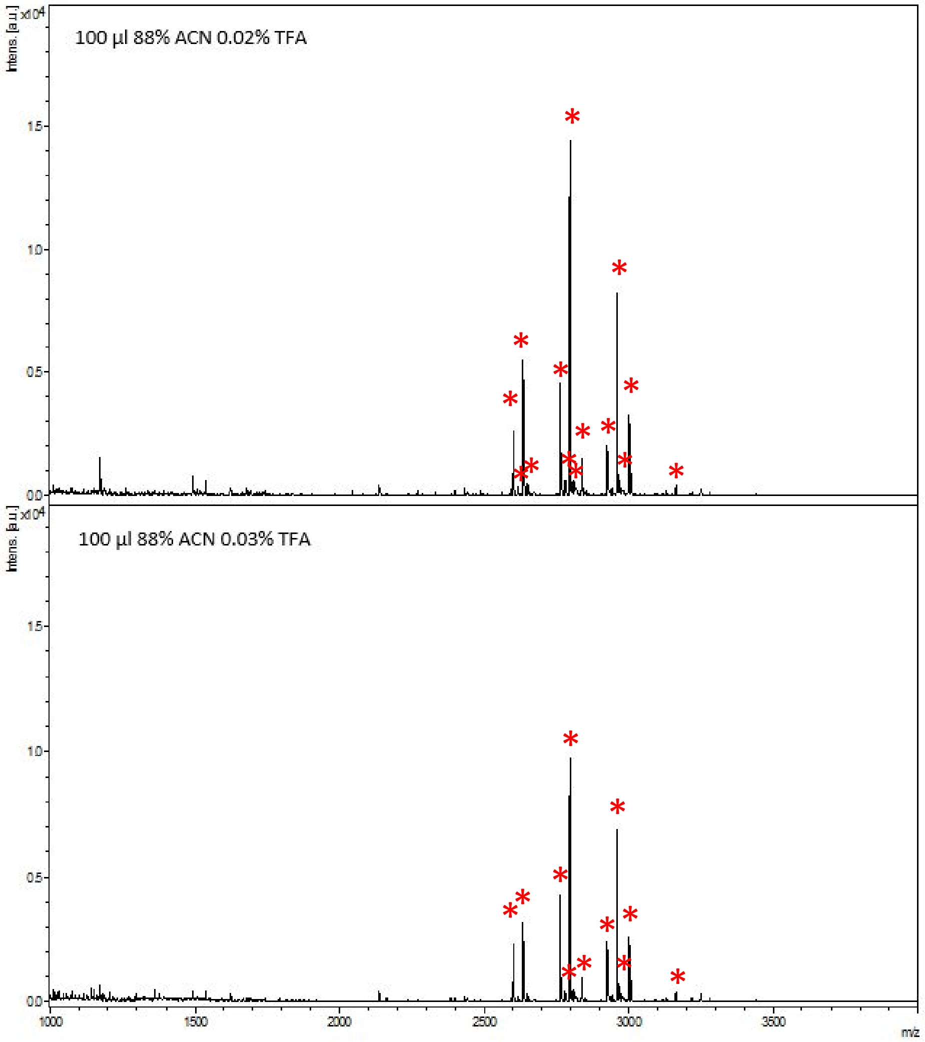

Figure S7. MALDI-TOF mass spectra of elution fractions after one washing with 100 µl of 88% ACN/0.02% TFA (top) and 100 µl 88% ACN/0.03% TFA (bottom). Glycopeptides are marked with red stars.

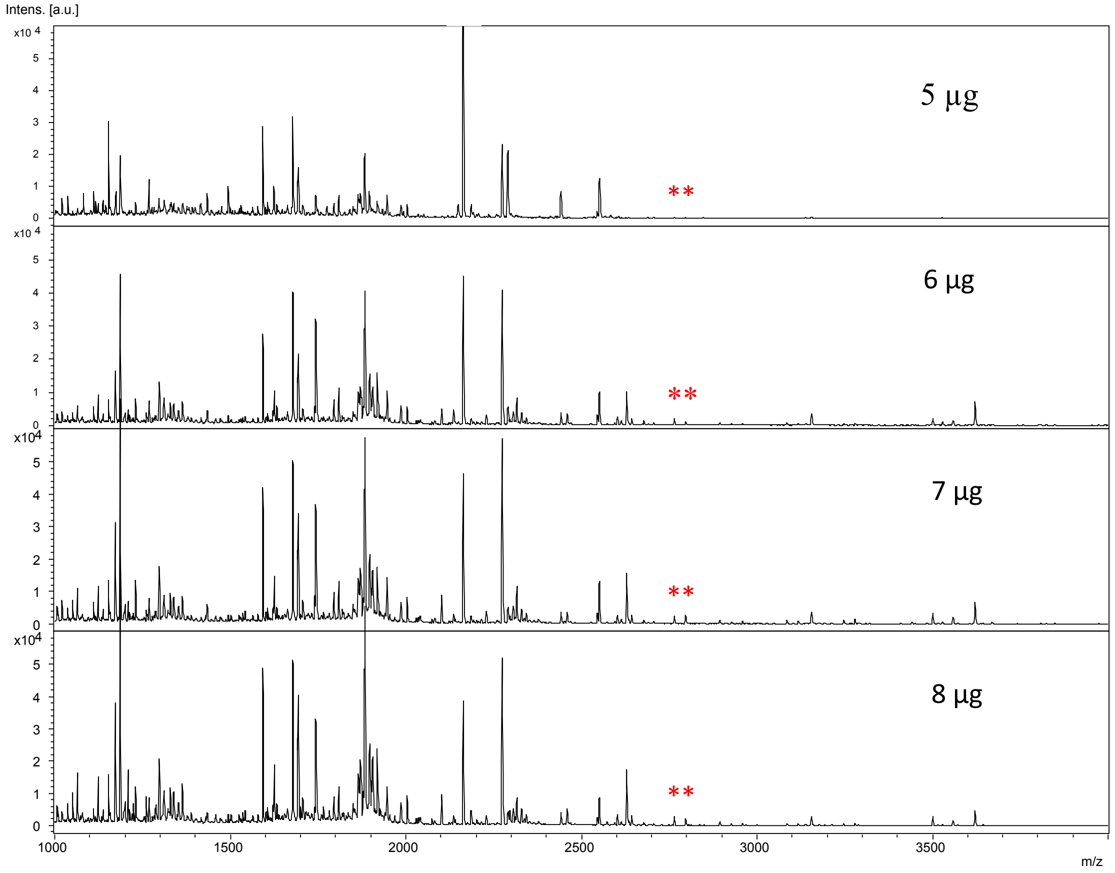

Figure S8. MALDI-TOF mass spectra of non-binding fractions of different amounts of IgG digest enriched with tips packed with 2 mg glass wool. Red stars mark glycopeptides with m/z 2764 and m/z 2796.

Table S4. Average S/N values of the six main glycopeptides detected in the elution fraction after one washing with 88% ACN/0.02% TFA or 88% ACN/0.03% TFA. n = 8, from two replicates. Data are presented as "mean ± standard deviation".

m/z	0.02% TFA	0.03% TFA
**2602**	51.38 ± 37.94	28.88 ± 14.58
**2634**	87.63 ± 58.23	56.75 ± 30.30
**2764**	105.13 ± 67.61	55.75 ± 28.97
**2796**	268.75 ± 147.77	173.63 ± 84.75
**2926**	53.75 ± 28.67	30.38 ± 17.41
**2958**	173.75 ± 83.81	113.88 ± 64.28

Table S5. IgG extracted from digested IgG standard samples. Glycan composition and structure of IgG glycopeptides enriched by glass wool and HILIC. Blue square: N-Acetylglucosamine (NAc), red triangle: fucose (Fuc), green circle: mannose (Hex), yellow circle: galactose (Hex), purple diamond: sialic acid (Sia). Detected: ✓. Non-detected: /.

Theoretical m/z	Glass wool	HILIC	Glycan composition	Glycan structure	Amino acid sequence
2430.967	✓	✓	(Hex)3 (NAc)3 (Fuc)1	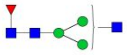	EEQYNSTYR (IgG1)
2487.988	/	✓	(Hex)3 (NAc)4	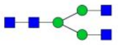	EEQYNSTYR (IgG1)
2602.056	✓	✓	(Hex)3 (NAc)4 (Fuc)1	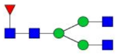	EEQFNSTFR (IgG2)
2618.051	✓	✓	(Hex)4 (NAc)4	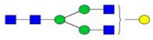	EEQFNSTFR (IgG2)
2634.046	✓	✓	(Hex)3 (NAc)4 (Fuc)1	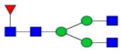	EEQYNSTYR (IgG1)
2650.041	✓	✓	(Hex)4 (NAc)4	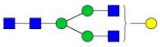	EEQYNSTYR (IgG1)
2764.109	✓	✓	(Hex)4 (NAc)4 (Fuc)1	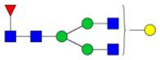	EEQFNSTFR (IgG2)
2780.104	✓	✓	(Hex)5 (NAc)4	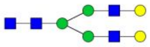	EEQFNSTFR (IgG2)
2796.099	✓	✓	(Hex)4 (NAc)4 (Fuc)1	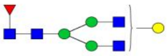	EEQYNSTYR (IgG1)
2805.135	✓	✓	(Hex)3 (NAc)5 (Fuc)1	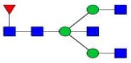	EEQFNSTFR (IgG2)
2812.094	✓	✓	(Hex)5 (NAc)4	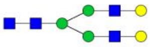	EEQYNSTYR (IgG1)
2837.125	✓	✓	(Hex)3 (NAc)5 (Fuc)1	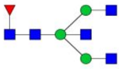	EEQYNSTYR (IgG1)
2926.162	✓	✓	(Hex)5 (NAc)4 (Fuc)1	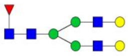	EEQFNSTFR (IgG2)
2958.152	✓	✓	(Hex)5 (NAc)4 (Fuc)1	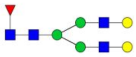	EEQYNSTYR (IgG1)
2967.188	✓	✓	(Hex)4 (NAc)5 (Fuc)1	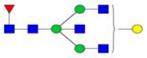	EEQFNSTFR (IgG2)
2999.178	✓	✓	(Hex)4 (NAc)5 (Fuc)1	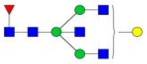	EEQYNSTYR (IgG1)
3161.231	✓	✓	(Hex)5 (NAc)5 (Fuc)1	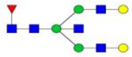	EEQYNSTYR (IgG1)
3249.247	✓	✓	(Hex)5 (NAc)4 (Fuc)1 (Sia)1	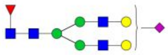	EEQYNSTYR (IgG1)
3278.396	✓	✓	(Hex)4 (NAc)4 (Fuc)1	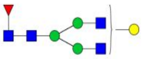	TKPREEEQYNSTYR (IgG1)

Table S6. Average S/N values of the six main glycopeptides enriched from IgG digests using glass wool and HILIC tips. n = 12, from three replicates. Data are presented as "mean ± standard deviation".

m/z	**HILIC (S/N)**	**Glass Wool (S/N)**			**Glass Wool (abundance in relation to m/z 2796))**		
	day 1<br>n=12<br>3 replicates	day 1<br>n=12<br>3 replicates	day 2<br>n=8<br>2 replicates	day 3<br>n=8<br>2 replicates	day 1<br>n=12<br>3 replicates	day 2<br>n=8<br>2 replicates	day 3<br>n=8<br>2 replicates
**2602**	172.8 ± 56.3	61.3 ± 23.8	81.1 ± 13.7	89.5 ± 28.3	0.18	0.15	0.19
**2634**	200.7 ± 78.6	131.3 ± 59.0	162.2 ± 93.4	159.5 ± 32.5	0.38	0.31	0.34
**2764**	214.9 ± 72.6	108.7 ± 41.6	152.4 ± 18.6	171.4 ± 54.1	0.31	0.29	0.37
**2796**	359.0 ± 132.3	347.7 ± 142.7	528.7 ± 156.5	466.6 ± 106.7	1.00	1.00	1.00
**2926**	83.1 ± 28.9	64.9 ± 21.2	103.6 ± 11.2	108.9 ± 37.3	0.19	0.20	0.23
**2958**	169.9 ± 65.0	250.1 ± 88.7	439.0 ± 111.6	352.9 ± 103.2	0.72	0.83	0.76

Table S7. Presence of six main glycopeptides enriched from IgG digests using glass wool at different concentrations. "+" illustrates an S/N value ≥ 6, "-"illustrates an S/N value < 6.

**IgG concentration**	**1.0 mg/mL**			**0.5 mg/mL**			**0.1 mg/mL**			**0.05 mg/mL**			**0.025 mg/mL**		
**Tip no**	1	2	3	1	2	3	1	2	3	1	2	3	1	2	3
**m/z**															
**2602**	+	+	+	+	+	+	+	+	+	+	+	+	+	-	+
**2634**	+	+	+	+	+	+	+	+	+	+	+	+	+	-	+
**2764**	+	+	+	+	+	+	+	+	+	+	+	+	+	-	+
**2796**	+	+	+	+	+	+	+	+	+	+	+	+	+	-	+
**2926**	+	+	+	+	+	+	+	+	+	+	+	+	-	-	-
**2958**	+	+	+	+	+	+	+	+	+	+	+	+	+	-	+

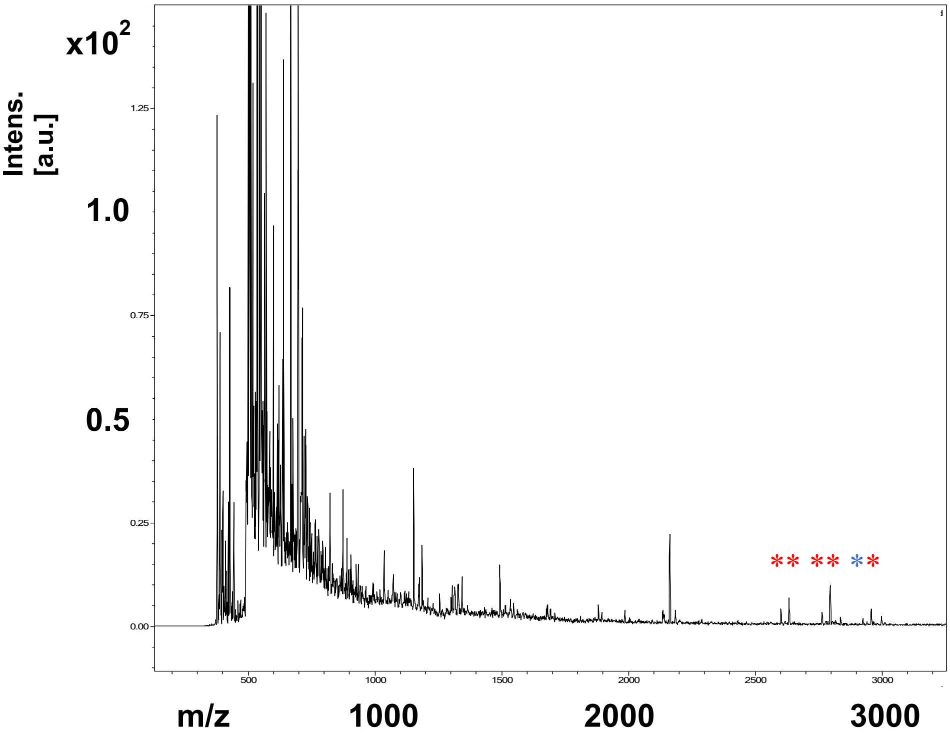

Figure S9. MALDI-TOF mass spectrum of elution fraction from IgG digest (0.025 mg/mL) enriched with a glass wool tip. Glycopeptides are marked with red stars, the blue star indicates m/z = 2926, which has an S/N value below 6.

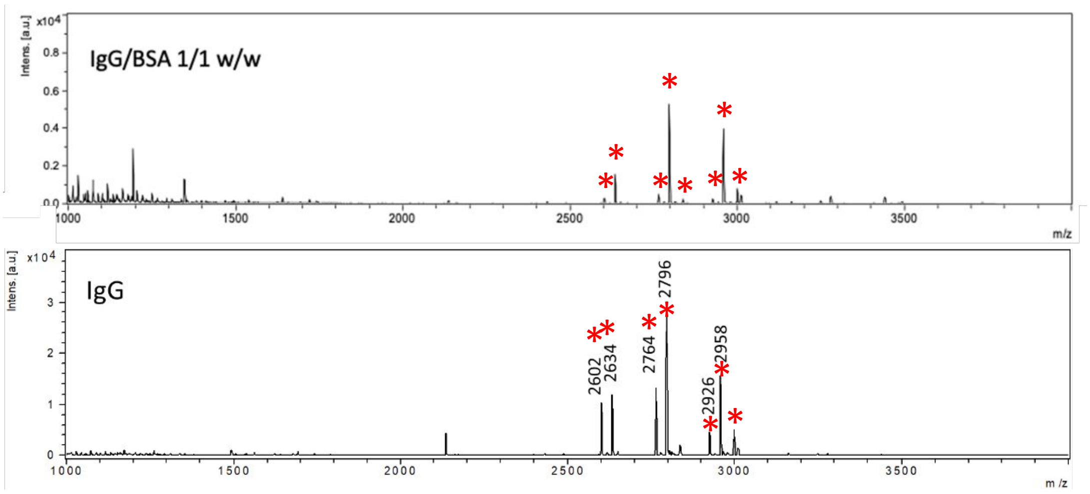

Figure S10. MALDI-TOF mass spectra of elution fractions of IgG digest with BSA digest (1/1 w/w) (top), and IgG digest (bottom). Glycopeptides are marked with red stars.

Table S8. Presence of three glycopeptides from elution fractions of IgG digest with BSA digest in three different ratios (1/3, 1/5 and 1/10). "+" illustrates an S/N value ≥ 6, "-" illustrates an S/N value < 6.

m/z	IgG:BSA 1:3			IgG:BSA 1:5			IgG:BSA 1:10		
	Tip no 1	Tip no 2	Tip no 3	Tip no 1	Tip no 2	Tip no 3	Tip no 1	Tip no 2	Tip no 3
**2634**	+	-	-	-	-	-	+	-	-
**2796**	+	+	-	+	+	+	+	-	+
**2958**	+	+	+	+	+	+	+	-	+

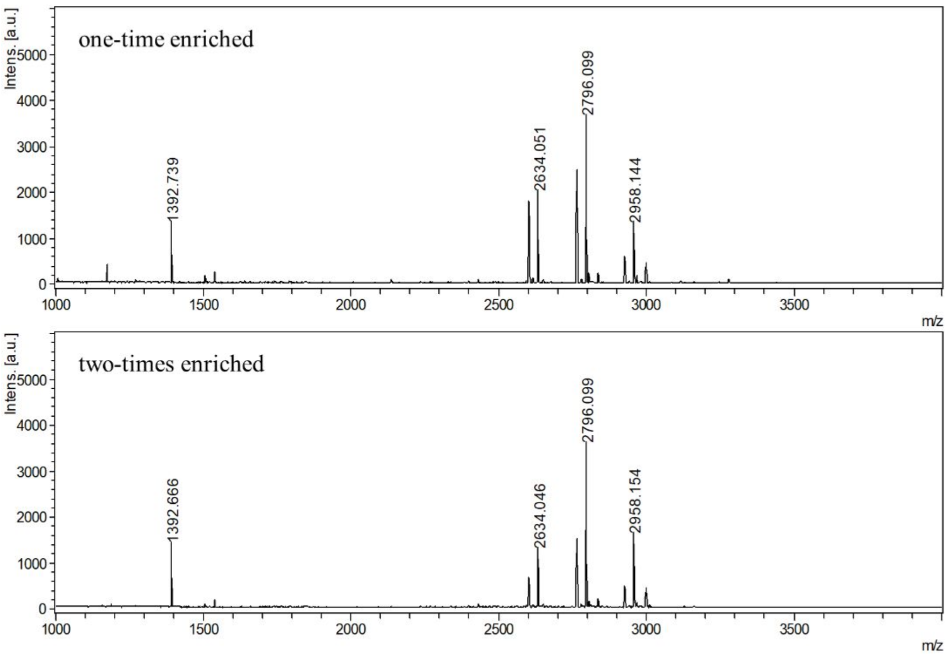

Figure S11. MALDI-TOF mass spectra of one and two enrichment cycles of IgG glycopeptides with the same amount of an IgG1 glycopeptide (EEQYN(GlcNAc)STYR) added as IS (50 ng/mL, *m/z* 1393). The volume ratio of IgG glycopeptides/IgG1 IS was 2/1.

Equation S1. The recovery for each glycopeptide was calculated for one and two enrichment cycles using the area ratios between the main IgG1 glycopeptides and the IgG1 IS.

Recovery = $(\text{Area}_{\text{two-times enriched glycopeptide}}/\text{Area}_{\text{IS}})/(\text{Area}_{\text{one-time enriched glycopeptide}}/\text{Area}_{\text{IS}}) \times 100\%$.

Table S9. Recovery calculated for the three main IgG1 glycopeptides using glass wool.

**Area values** of **one-time** enriched main IgG1 glycopeptides and IS															
IS	1016	591	1168	1014	881	1182	548	568	1469	897	1651	964			
2634	1128	597	1346	979	816	1093	525	526	1548	868	1560	940			
2796	2576	1323	2876	2162	1793	2453	1135	1058	3402	1910	3188	2014			
2958	756	413	879	684	557	743	350	375	1077	593	1117	619			
**Area ratios** between the **one-time** enriched main IgG1 glycopeptides and the IS													**Mean**	**RSD (%)**	
2634/IS	1.11	1.01	1.15	0.97	0.93	0.92	0.96	0.93	1.05	0.97	0.94	0.98	**0.99±0.07**	**7.5**	
2796/IS	2.54	2.24	2.46	2.13	2.04	2.08	2.07	1.86	2.32	2.13	1.93	2.09	**2.16±0.20**	**9.5**	
2958/IS	0.74	0.70	0.75	0.67	0.63	0.63	0.64	0.66	0.73	0.66	0.68	0.64	**0.68±0.04**	**6.4**	
**Area values** of **two-times** enriched main IgG1 glycopeptides and IS															
IS	1081	1108	951	1922	3616	1482	1611	3525	3852	2445	2680	1608			
2634	693	653	670	1038	1983	841	876	2028	2360	1403	1535	952			
2796	2007	2020	1920	3112	5975	2349	2817	5917	6718	3889	4664	3035			
2958	780	767	700	1229	2353	986	1082	2284	2630	1648	1772	1152			
**Area ratios** between the **two-times** enriched main IgG1 glycopeptides and the IS													**Mean**	**RSD (%)**	**Recovery (%)**
2634/IS	0.64	0.59	0.70	0.54	0.55	0.57	0.54	0.58	0.61	0.57	0.57	0.59	**0.59±0.05**	**7.8**	**59.27**
2796/IS	1.86	1.82	2.02	1.62	1.65	1.59	1.75	1.68	1.74	1.59	1.74	1.89	**1.75±0.13**	**7.6**	**80.94**
2958/IS	0.72	0.69	0.74	0.64	0.65	0.67	0.67	0.65	0.68	0.67	0.66	0.72	**0.68±0.03**	**4.7**	**100.20**

Table S10. Area values and proportion of G0F, G1F, and G2F for IgG1 and IgG2 from patient and reference samples.

**Patient**	**Area of the mix main glycopeptides**														
	m/z	spot 1	spot 2	spot 3	spot 4	spot 5	spot 6	spot 7	spot 8	spot 9	spot 10	spot 11	spot 12		
**IgG2**	2602	1304	1257	829	1299	1552	1550	1062	1001	1321	1688	959	1065		
	2764	2210	2121	1388	2228	2127	2213	1453	1349	1764	2167	1290	1451		
	2926	657	651	421	712	581	603	413	396	499	581	363	416		
**IgG1**	2634	995	998	458	836	1203	1359	875	601	927	1293	538	476		
	2796	3578	3691	1545	3193	3819	4634	2780	1996	2996	3852	1656	1447		
	2958	2079	2186	882	1874	2238	2761	1653	1141	1732	2200	953	851		
	**proportion of G0F, G1F, and G2F (%)**													**mean**	**std**
**IgG2**	G0F	31.26	31.20	31.43	30.64	36.43	35.50	36.27	36.45	36.86	38.05	36.72	36.32	**34.76**	**2.75**
	G1F	52.98	52.64	52.62	52.56	49.93	50.69	49.62	49.13	49.22	48.85	49.39	49.49	**50.59**	**1.62**
	G2F	15.75	16.16	15.96	16.80	13.64	13.81	14.11	14.42	13.92	13.10	13.90	14.19	**14.65**	**1.19**
**IgG1**	G0F	14.96	14.52	15.88	14.16	16.57	15.52	16.48	16.08	16.39	17.60	17.10	17.16	**16.04**	**1.08**
	G1F	53.79	53.69	53.55	54.09	52.60	52.94	52.37	53.40	52.98	52.44	52.62	52.16	**53.05**	**0.63**
	G2F	31.25	31.80	30.57	31.75	30.83	31.54	31.14	30.52	30.63	29.95	30.28	30.68	**30.91**	**0.59**

**Reference**	**Area of the mix main glycopeptides**														
	m/z	spot 1	spot 2	spot 3	spot 4	spot 5	spot 6	spot 7	spot 8	spot 9	spot 10	spot 11	spot 12		
**IgG2**	2602	677	1339	875	1336	929	1438	402	952	1359	719	634	897		
	2764	1251	2406	1635	2637	1580	2561	611	1594	2035	1148	978	1423		
	2926	743	1327	908	1558	833	1471	447	1153	1229	707	684	1002		
**IgG1**	2634	672	1350	1542	1096	457	618	529	875	1740	570	756	1036		
	2796	2260	4219	4615	3830	1423	2009	1286	2137	4372	1425	1815	2610		
	2958	2014	3688	4132	3689	1247	1766	1286	2141	3783	1232	1665	2303		
	**proportion of G0F, G1F, and G2F (%)**													**mean**	**std**
**IgG2**	G0F	25.35	26.40	25.60	24.15	27.80	26.29	27.53	25.74	29.40	27.93	27.61	27.00	**26.73**	**1.42**
	G1F	46.84	47.44	47.83	47.68	47.28	46.82	41.85	43.09	44.02	44.60	42.60	42.84	**45.24**	**2.29**
	G2F	27.82	26.16	26.57	28.17	24.93	26.89	30.62	31.17	26.58	27.47	29.79	30.16	**28.03**	**1.98**
**IgG1**	G0F	13.59	14.58	14.99	12.72	14.61	14.07	17.06	16.98	17.58	17.66	17.85	17.41	**15.76**	**1.84**
	G1F	45.69	45.58	44.85	44.46	45.51	45.73	41.47	41.47	44.18	44.16	42.85	43.87	**44.15**	**1.52**
	G2F	40.72	39.84	40.16	42.82	39.88	40.20	41.47	41.55	38.23	38.18	39.31	38.71	**40.09**	**1.40**
